# High expression of *EZR* (ezrin) gene is correlated with the poor overall survival of breast cancer patients

**DOI:** 10.1111/1759-7714.13174

**Published:** 2019-08-26

**Authors:** Rongju Zhang, Shaohui Zhang, Rongge Xing, Qin Zhang

**Affiliations:** ^1^ Department of Pathology Cangzhou Central Hospital Changzhou China; ^2^ Department of Orthopaedics Cangzhou Hospital of Integrated Traditional Chinese and Western Medicine of Hebei Province Cangzhou China; ^3^ Department of Thyroid and Breast Surgery Cangzhou Central Hospital Changzhou China

**Keywords:** Breast cancer, *EZR* gene, immunohistochemistry, prognosis

## Abstract

**Background:**

To evaluate the *EZR* (ezrin) gene expression in breast cancer and correlation with the prognosis through bioinformatics analysis and immunohistochemistry assay.

**Methods:**

*EZR* gene expression in breast cancer and corresponding normal breast tissue was compared in the TCGA database. Protein‐protein interaction (PPI) network relevant EZR was established through the STRING database. The correlation between *EZR* expression and prognosis of breast cancer was analyzed by the log‐rank analysis from the TCGA. Ezrin protein (coded by EZR) expression was also examined by immunohistochemistry assay in 120 breast cancer patients.

**Results:**

*EZR* expression level in tumor tissue was significantly upregulated compared to that of normal breast tissue of breast cancer patients (*P* < 0.05). In the PPI analysis, there were 51 nodes and 455 edges in the network. The top 10 hub genes of the network were identified. High expression of EZR mRNA was correlated with poor overall survival (OS) of the breast cancer patients (HR = 1.40, *P* = 0.038). However, the disease‐free survival (DFS) of breast cancer patients did not correlate with the EZR mRNA level (HR = 0.86, *P* = 0.44). The ezrin protein expression was positive with uniform brown‐yellow granules in the cell membrane, cavity surface and cytoplasm of the breast cancer cells. Of the included 120 cancer samples, 98 cases were positive for ezrin expression and 22 were negative. No correlation was found between ezrin expression site and patients’ clinicopathological features.

**Conclusion:**

EZR is upregulated in breast cancer and can be used as potential biomarker for overall survival.

## Introduction

Breast cancer is known as one of the leading causes of malignant carcinoma‐related death globally.^1^, ^2^ In recent years, the incidence of breast cancer had gradually increased, surpassing cervical cancer and becoming the highest incidence of malignant tumors for women.^3^ It has been estimated that around 1.2 million breast cancer patients in the world are diagnosed each year.^4^ In China, the incidence of breast cancer is about 23/100000, accounting for 7–10% of all malignant tumors.^5^ However, the molecular mechanism of breast cancer remains unclear.

Studies have demonstrated that *EZR* gene is upregulated in malignant solid carcinoma and is correlated with patients’ prognosis.^6^, ^7^ However, correlation between *EZR* gene expression in breast cancer and its correlation with patients’ survival is not fully understood. In recent years, with the development of cancer databases, more and more studies have been published in the aspects of deep data mining in the database through bioinformatics analysis.^8^, ^9^ In the present work, we investigated the *EZR* gene expression in breast cancer, function enrichment and correlation with the prognosis through bioinformatics analysis and immunohistochemistry assay in order to provide a greater understanding of the *EZR* gene in breast cancer.

## Methods

### *EZR* expression in breast cancer and corresponding normal tissue

*EZR* expression in breast cancer and corresponding normal tissue was identified from The Cancer Genome Atlas (TCGA) database through UALCAN online data mining website (http://ualcan.path.uab.edu/index.html). *EZR* expression in pan‐cancer and corresponding normal tissue was also evaluated.

### PPI network construction and hub genes identification

The search tool for the retrieval of interacting genes (STRING) (http://string-db.org/cgi/input.pl) was used to constructed the protein‐protein network (PPI) of *EZR* and relevant genes to evaluate the correlation between *EZR* expression and breast cancer. To screen the stable correlation between *EZR* and relevant genes, the maximum number of interactors was restricted to no more than 50 and minimum required interaction score of 0.4. Ten hub genes were identified by Cytoscape (Cytoscape_v3.6.1, https://cytoscape.org/).

### Gene ontology (Go) and Kyoto encyclopedia of genes and genomes (KEGG) enrichment

The biological function and KEGG pathway^10^, ^11^, ^12^ of *EZR* and relevant genes in the network was enriched. For GO enrichment,^13^ there was three aspects including biological process (BP), cellular component (CC) and molecular function (MF). The enrichment was demonstrated by the bubble chart which shows the gene ratio, Q‐value, gene count and gene description.

### Survival analysis

According to the median expression level of *EZR* in breast cancer tissues, the patients were divided into high and low expression groups. Survival analysis (Kaplan‐Meier curve) and log‐rank test were used to calculate the difference of total survival (OS) and disease‐free progression survival (DFS) between high and low expression groups through the log‐rank analysis by the online data analysis tool GEPIA. GEPIA is a newly developed interactive web server for analyzing the RNA sequencing expression data of 9736 tumors and 8587 normal samples from the TCGA and the GTEx projects, using a standard processing pipeline. GEPIA provides customizable functions such as tumor/normal differential expression analysis, profiling according to cancer types or pathological stages, patient survival analysis, similar gene detection, correlation analysis and dimensionality reduction analysis.

### Immunohistochemistry assay

A total of 120 cancer tissue samples were included in the present work which was approved by the Ethical Committee of Cangzhou Central Hospital. All patients gave their signed informed consent. Ezrin protein (coded by EZR) expression was examined by immunohistochemistry assay in 120 breast cancer patient and ezrin expression and patients’ clinical characteristics were analyzed.

## Results

### EZR expression in breast cancer

**Figure 1:**
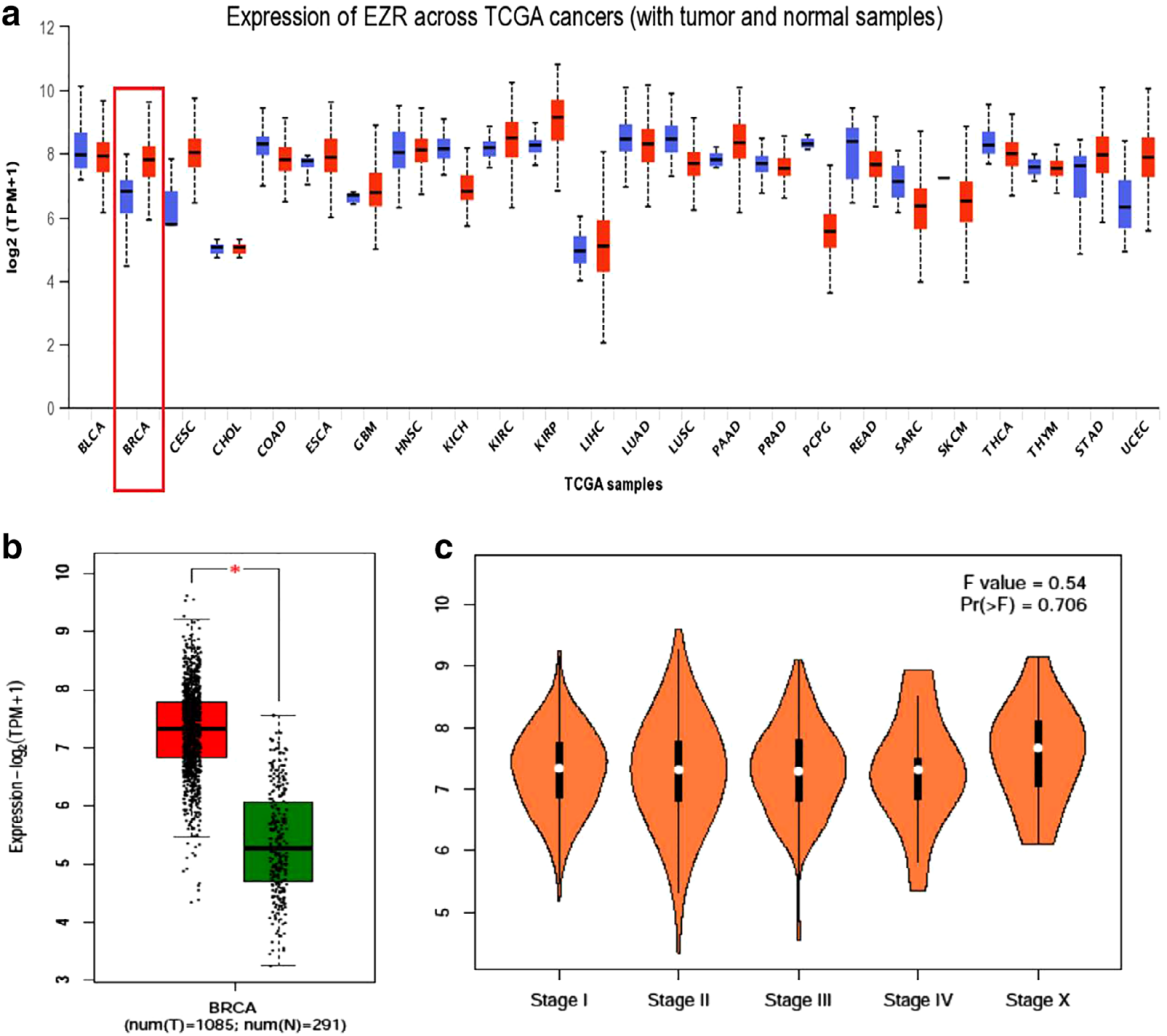
*EZR* expression in breast cancer and other malignant tumors (**a**) EZR mRNA expression in pan‐cancers and corresponding normal tissue; (**b**) scatter plot of *EZR* expression breast cancer and normal breast tissue; (**c**) *EZR* expression in breast cancer (stages I–X).

EZR mRNA expression level in tumor tissue was significantly upregulated compared to that of normal breast tissue of breast cancer patients (*P* < 0.05). However, EZR mRNA expression level was not significantly different among different clinical stages (Fig 1).

### PPI analyses and hub genes identification

**Figure 2:**
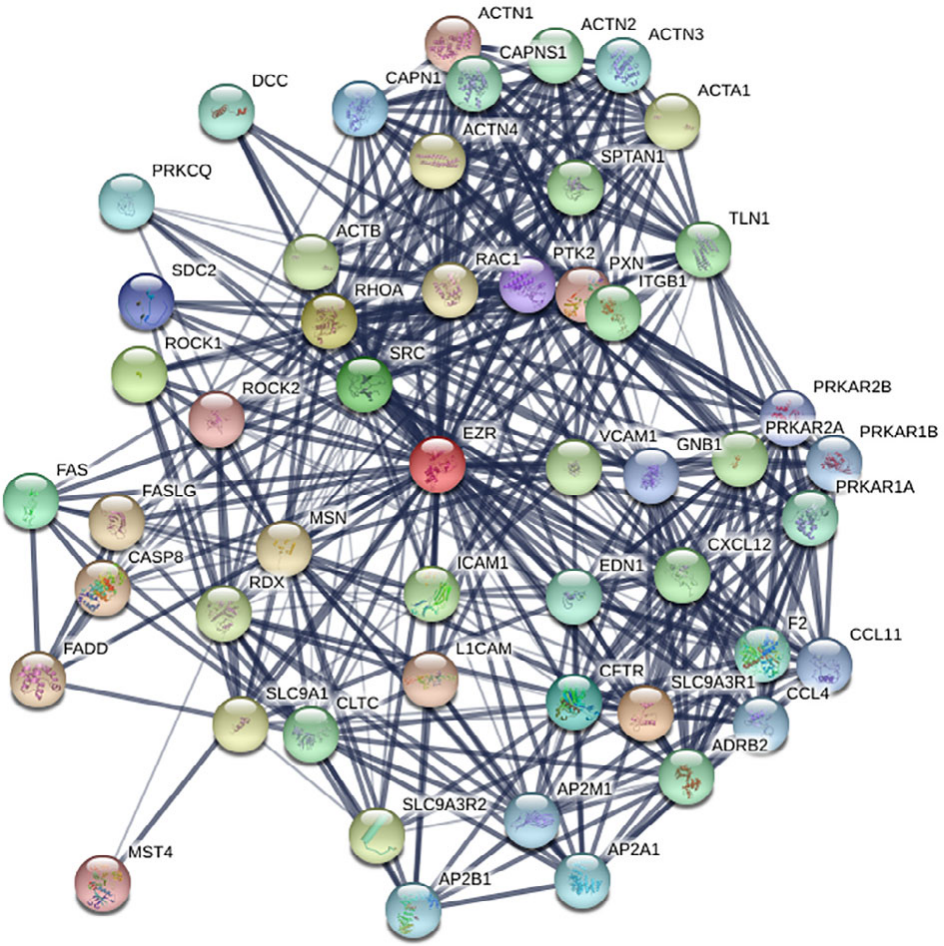
Protein‐protein network analysis of *EZR* in breast cancer.

**Figure 3:**
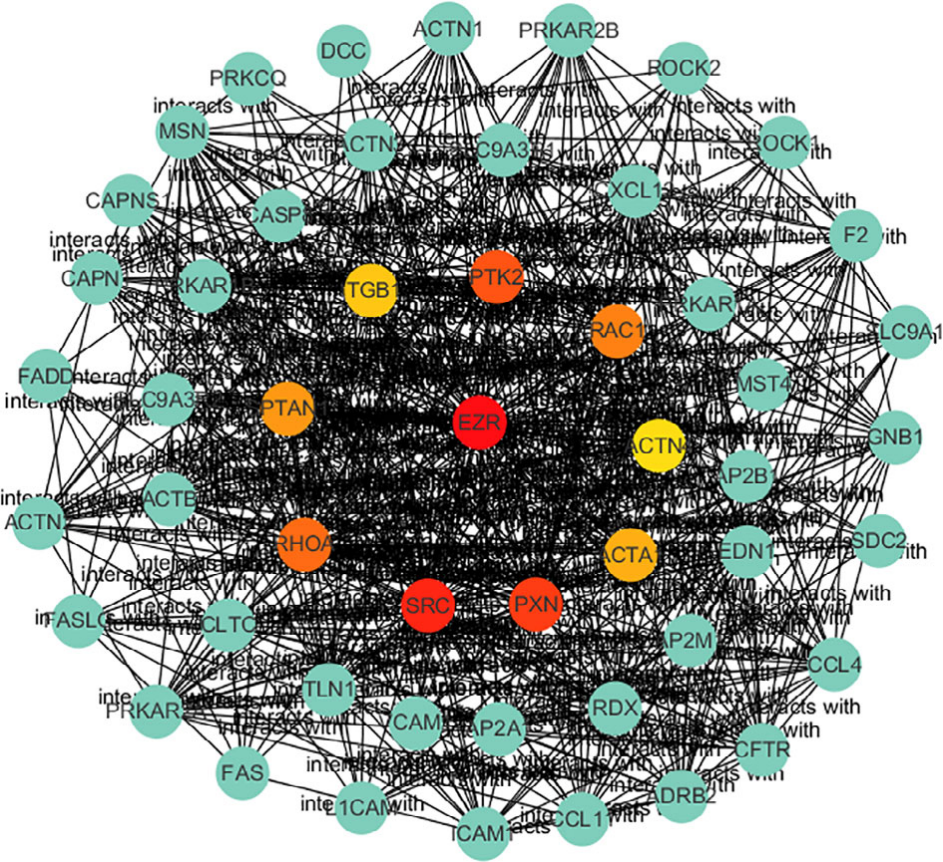
Hub gene identification of the protein‐protein network relevant to EZR in breast cancer.

To further investigate the role of the *EZR* gene in the development of breast cancer, we constructed the protein‐protein network by STRING to identify the interaction between the relevant genes. There were 51 nodes and 455 edges in the network (Fig 2). The top 10 hub genes of the network were further screened through Cytoscape (Fig 3).

### GO and KEGG analysis

**Figure 4:**
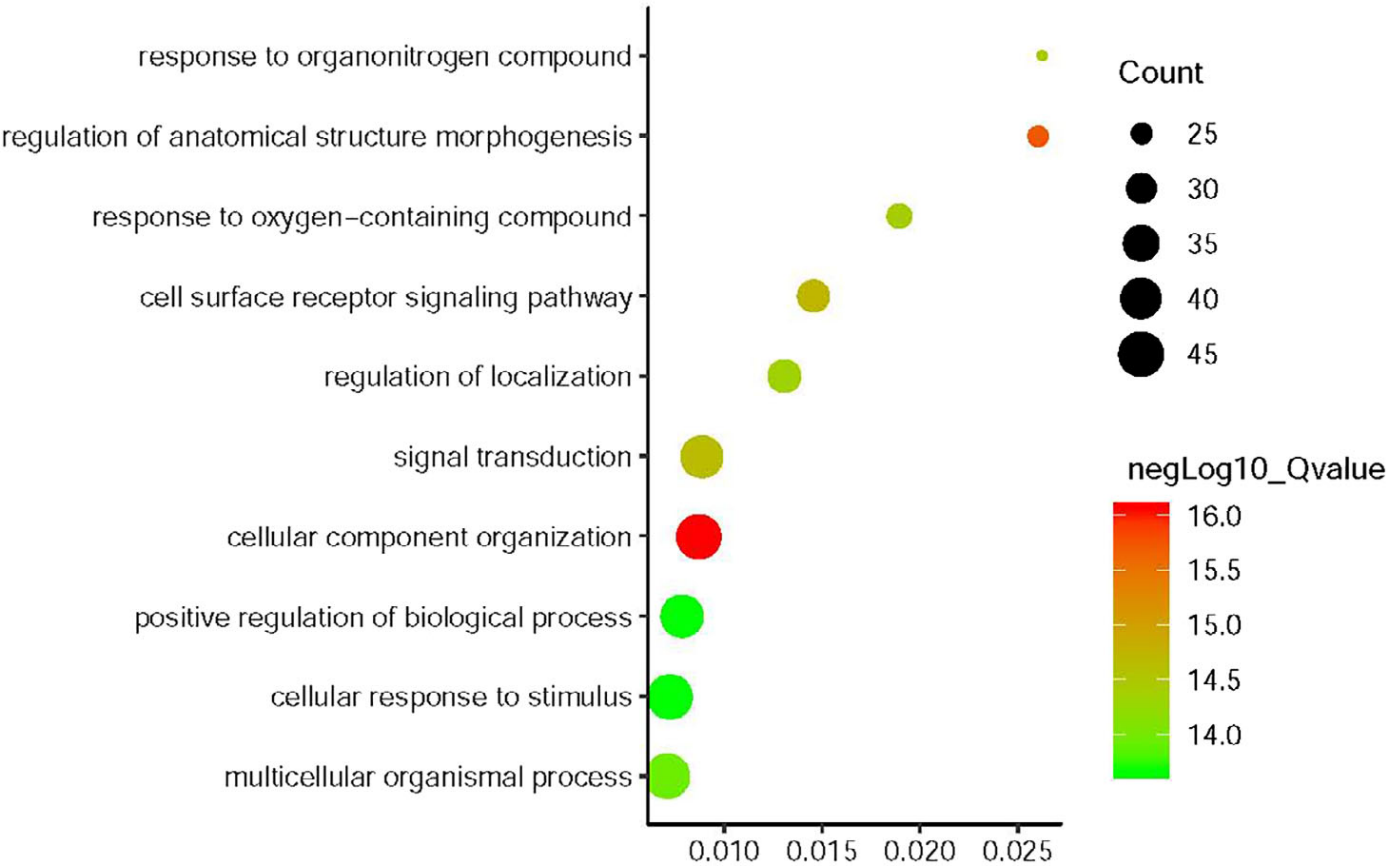
Go enrichment of *EZR* and relevant genes in the aspects of biological process.

**Figure 5:**
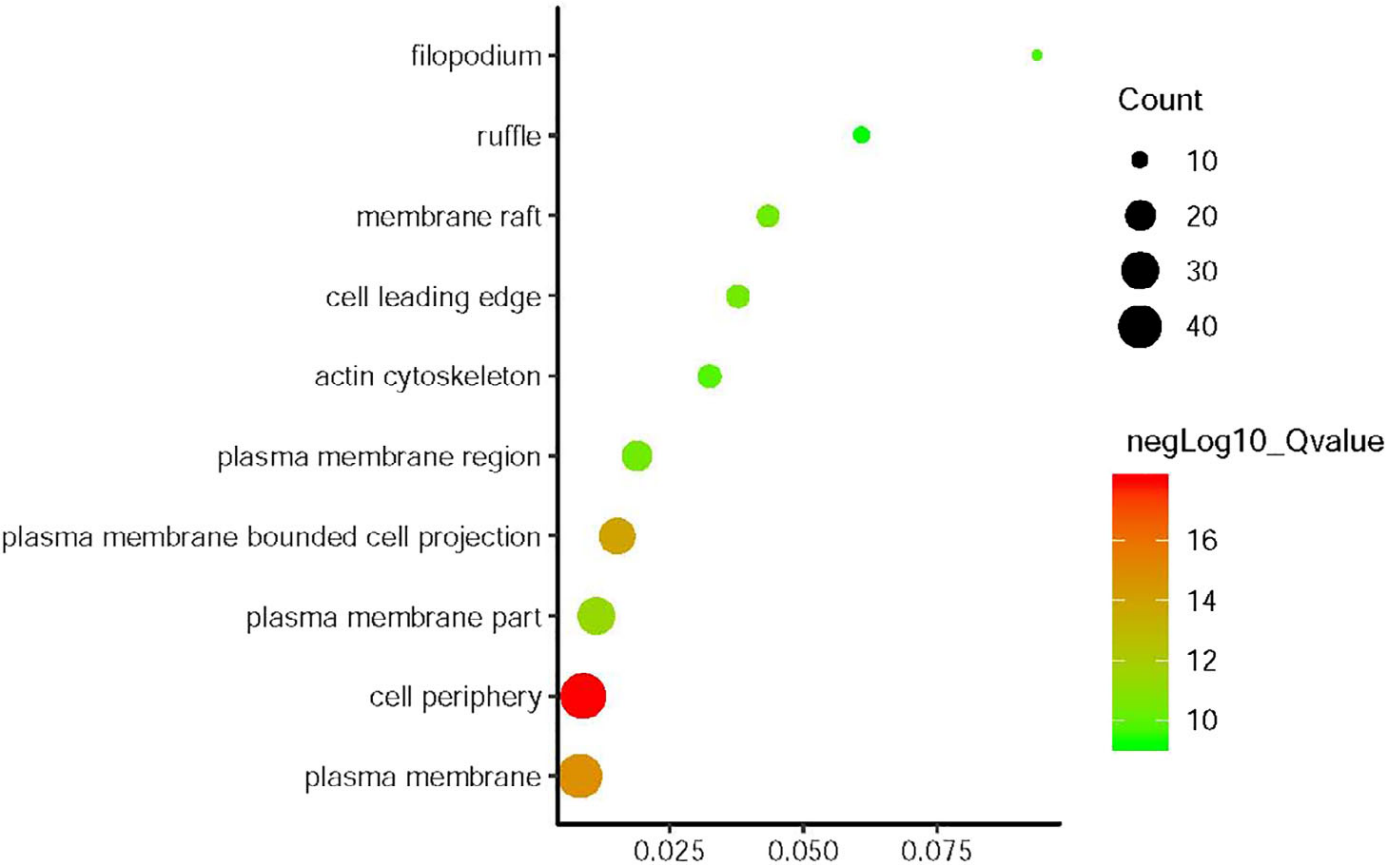
Go enrichment of *EZR* and relevant genes in the aspects of cellular component.

**Figure 6:**
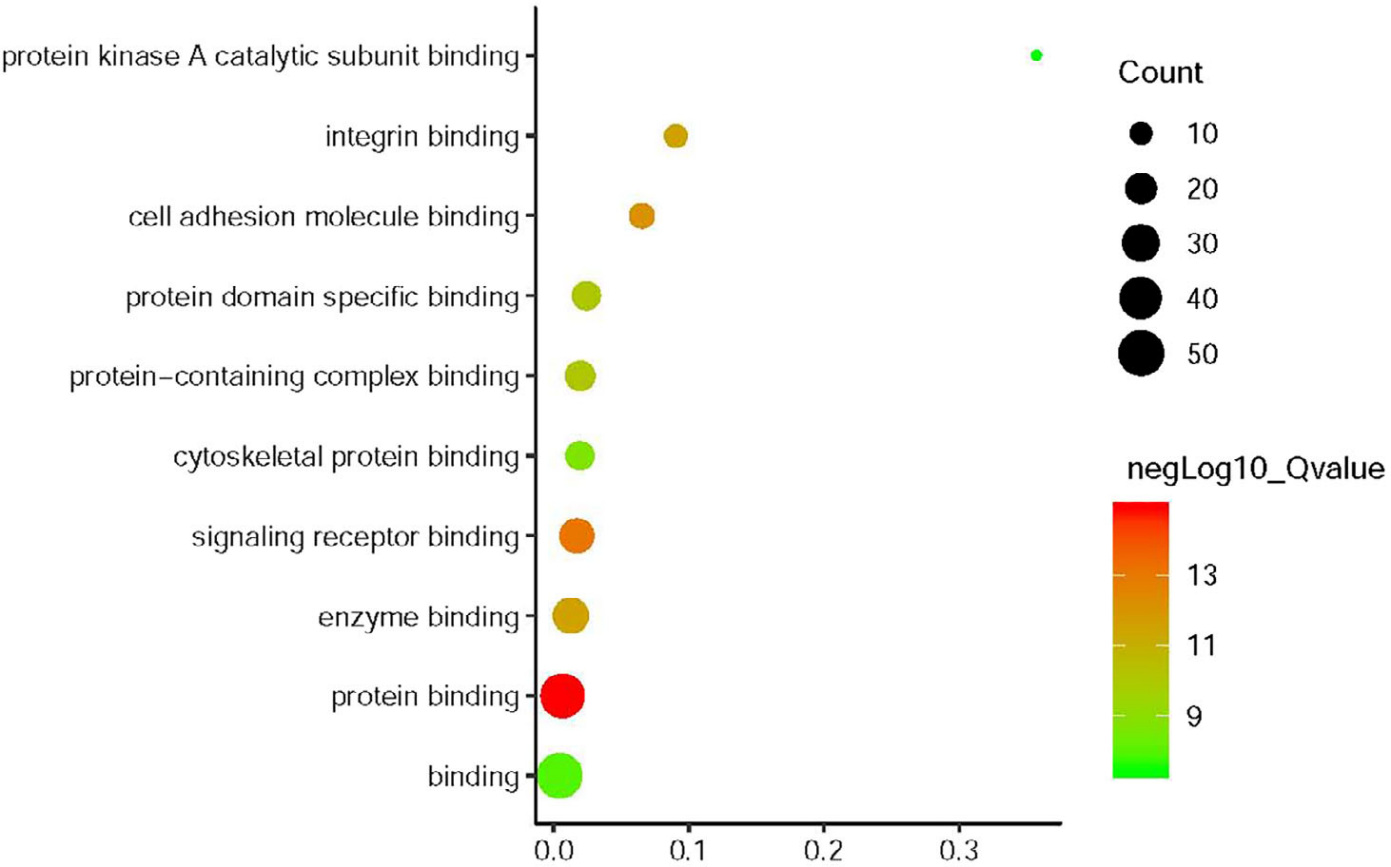
Go enrichment of *EZR* and relevant genes in the aspects of molecular function.

**Figure 7:**
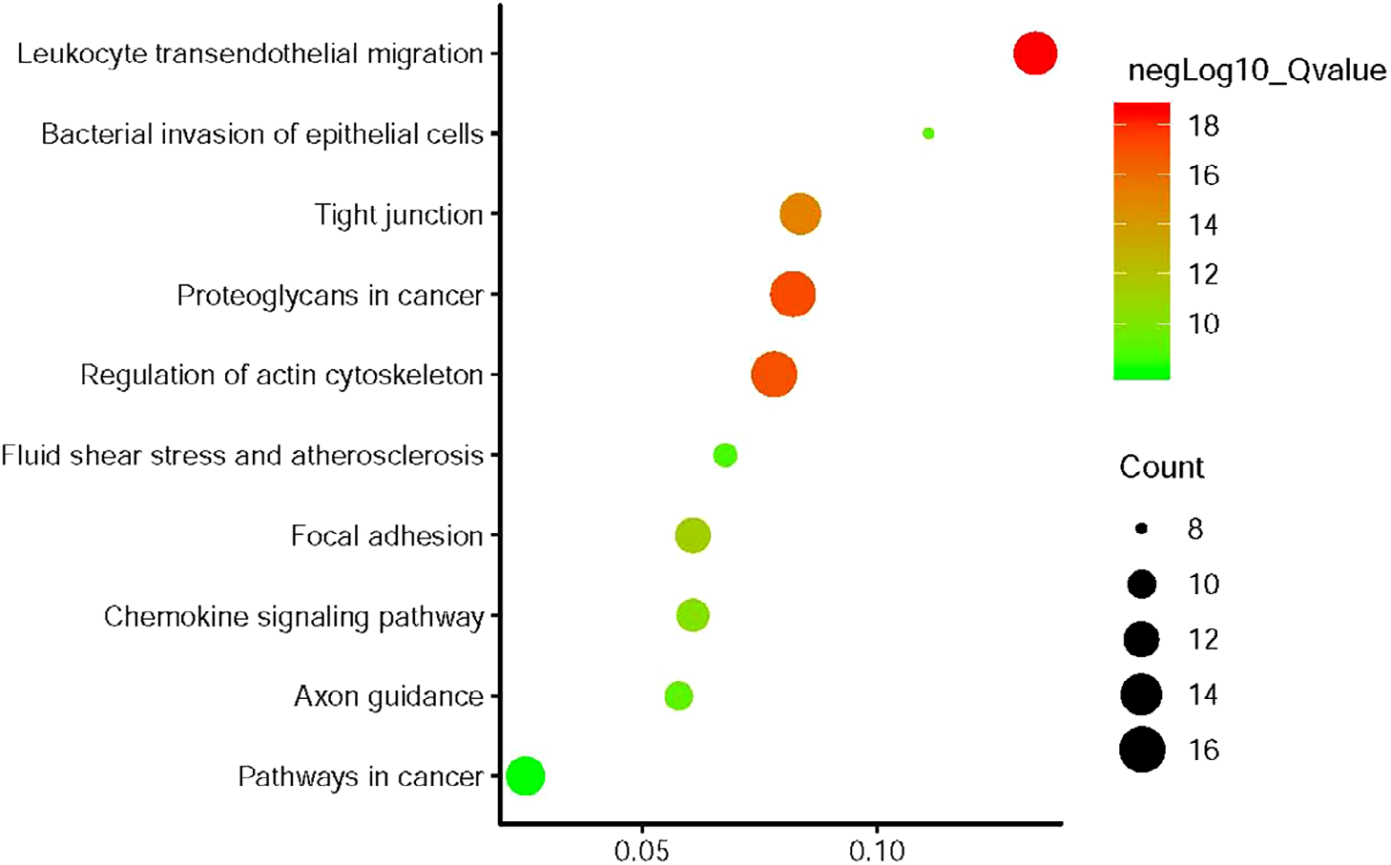
KEGG enrichment of *EZR* and relevant genes.

*EZR* and network relevant genes were mainly enriched in the cellular component organization, regulation of anatomical structure morphogenesis, cell surface receptor signaling pathway and etc for the biological process, Fig 4, Table 1. For cellular component, the genes enriched in cell periphery, plasma membrane, plasma membrane bounded cell projection (Fig 5, Table 2) For molecular function, the *EZR* and network relevant genes were mainly enriched in protein binding, signaling receptor binding, and cell adhesion molecule binding (Fig 6, Table 3 **)**. KEGG pathway analysis indicated that the leukocyte transendothelial migration, proteoglycans in cancer, regulation of actin cytoskeleton were included in the KEGG pathway (Fig 7, Table 4 **)**.

**Table 1:** Biological process enrichment of *EZR* and relevant genes

Gene ratio	Q‐value	Count	Description
45/5163	8.76E‐17	45	Cellular component organization
25/961	1.87E‐16	25	Regulation of anatomical structure morphogenesis
32/2198	1.87E‐15	32	Cell surface receptor signaling pathway
42/4738	2.29E‐15	42	Signal transduction
23/876	3.28E‐15	23	Response to organonitrogen compound
27/1427	3.90E‐15	27	Response to oxygen‐containing compound
33/2524	4.59E‐15	33	Regulation of localization
46/6507	1.13E‐14	46	Multicellular organismal process
43/5459	2.13E‐14	43	Positive regulation of biological process
45/6212	2.13E‐14	45	Cellular response to stimulus

**Table 2:** Cellular component enrichment of *EZR* and relevant genes

Gene ratio	Count	Q‐value	Description
46/5254	46	1.09E‐18	Cell periphery
43/5159	43	1.44E‐15	Plasma membrane
29/1900	29	1.04E‐14	Plasma membrane bounded cell projection
30/2651	30	4.21E‐12	Plasma membrane part
14/371	14	3.79E‐11	Cell leading edge
13/300	13	4.15E‐11	Membrane raft
20/1061	20	4.15E‐11	Plasma membrane region
14/432	14	1.50E‐10	Actin cytoskeleton
9/96	9	1.83E‐10	Filopodium
10/164	10	5.71E‐10	Ruffle

**Table 3:** Molecular function enrichment of the EZR and relevant genes

Gene ratio	Count	Q value	Description
47/6605	47	1.25E‐15	Protein binding
26/1513	26	8.85E‐14	Signaling receptor binding
13/200	13	7.33E‐13	Cell adhesion molecule binding
11/122	11	2.84E‐12	Integrin binding
28/2197	28	2.84E‐12	Enzyme binding
17/706	17	8.90E‐11	Protein domain specific binding
19/968	19	8.92E‐11	Protein‐containing complex binding
17/882	17	2.09E‐09	Cytoskeletal protein binding
50/11878	50	1.14E‐08	Binding
5/14	5	3.61E‐08	Protein kinase A catalytic subunit binding

**Table 4:** KEGG pathway enrichment of *EZR* and relevant genes

Gene ratio	Count	Q‐value	Description
15/112	15	2.52E‐19	Leukocyte transendothelial migration
16/195	16	7.12E‐18	Proteoglycans in cancer
16/205	16	1.01E‐17	Regulation of actin cytoskeleton
14/167	14	7.15E‐16	Tight junction
12/197	12	5.25E‐12	Focal adhesion
11/181	11	5.11E‐11	Chemokine signaling pathway
8/72	8	6.45E‐10	Bacterial invasion of epithelial cells
10/173	10	6.84E‐10	Axon guidance
9/133	9	1.69E‐09	Fluid shear stress and atherosclerosis
13/515	13	9.61E‐09	Pathways in cancer

### Survival analysis

**Figure 8:**
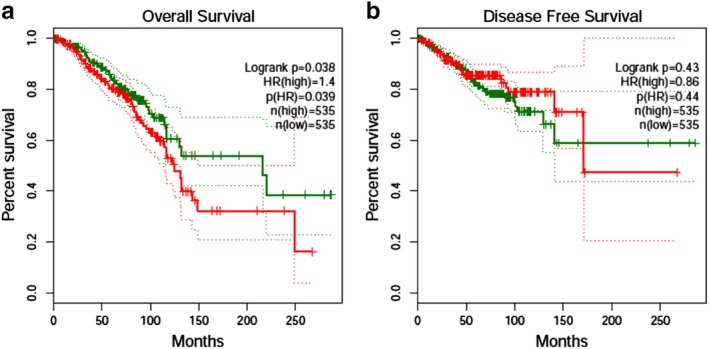
Survival curve for *EZR* high and low expression group of breast cancer (**a**) Overall survival comparison between *EZR* high (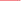) and low (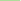) expression group; (**b**) disease‐free survival comparison between *EZR* high (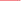) and low (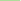) expression groups.

**Figure 9:**
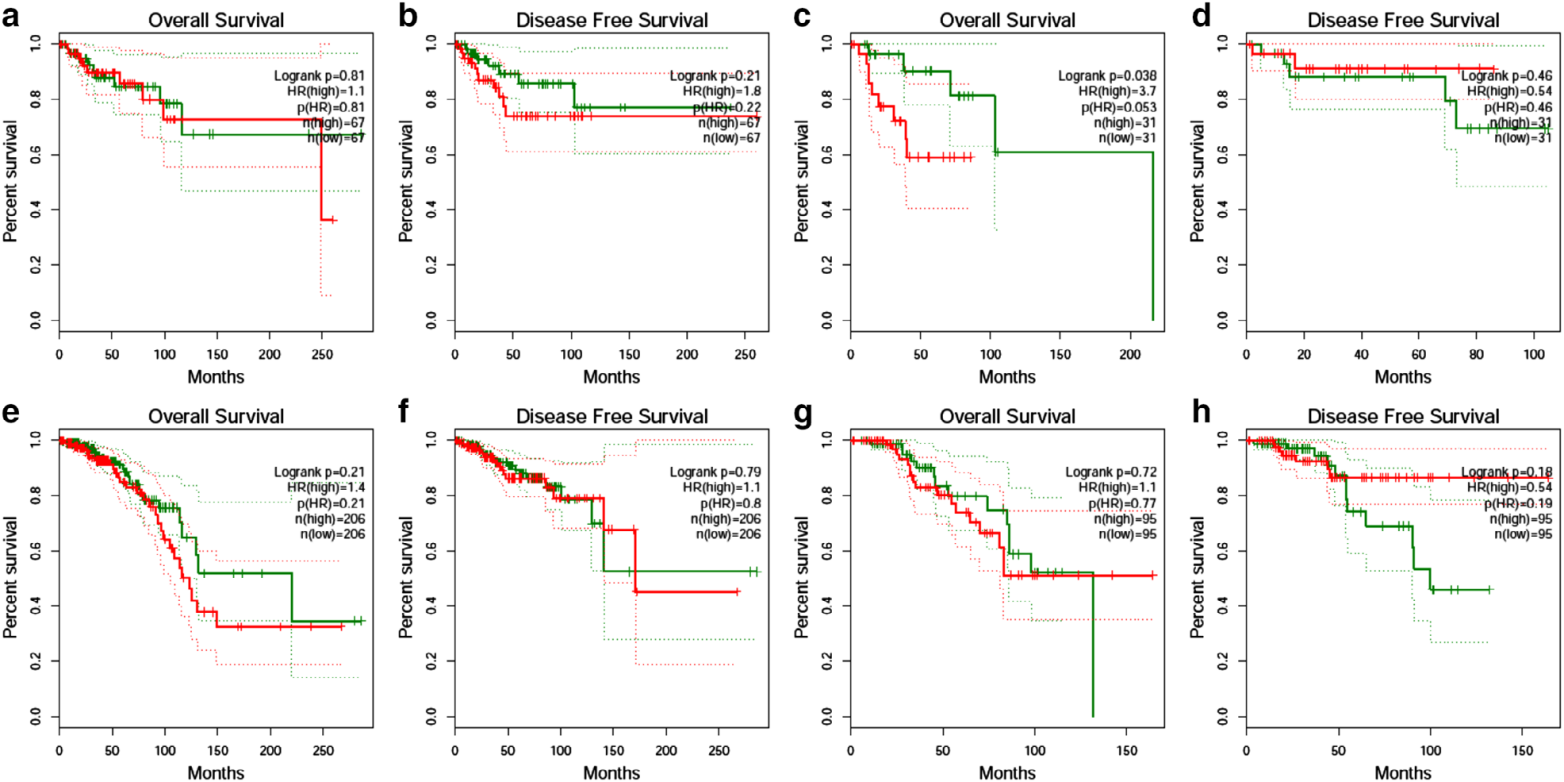
Survival curve for *EZR* high and low expression group of subgroup breast cancer (**a**) Overall survival of triple negative breast cancer; (**b**) disease‐free survival of triple negative breast cancer; (**c**) overall survival of HER2+ nonluminal breast cancer; (**d**) disease‐free survival of HER2+ nonluminal breast cancer; (**e**) overall survival of luminal A breast cancer; (**f**) disease‐free survival of luminal A breast cancer; (**g**) overall survival of luminal B breast cancer; (**h**) disease‐free survival of luminal A breast cancer.

High expression of EZR mRNA was correlated with poor overall survival of the breast cancer patients (HR = 1.40, *P* = 0.038), Fig 8a. However, the disease‐free survival (DFS) of breast cancer patients was not correlated with the EZR mRNA level (HR = 0.86, *P* = 0.44), Fig 8b. Subgroup analysis showed that the overall survival of HER2+ nonluminal breast cancer was significantly decreased in the *EZR* high expression group compared to the low expression group (HR = 3.7, *P* = 0.038) (Fig 9 **)**.

### *EZR* expression and clinicopathological features

**Figure 10:**
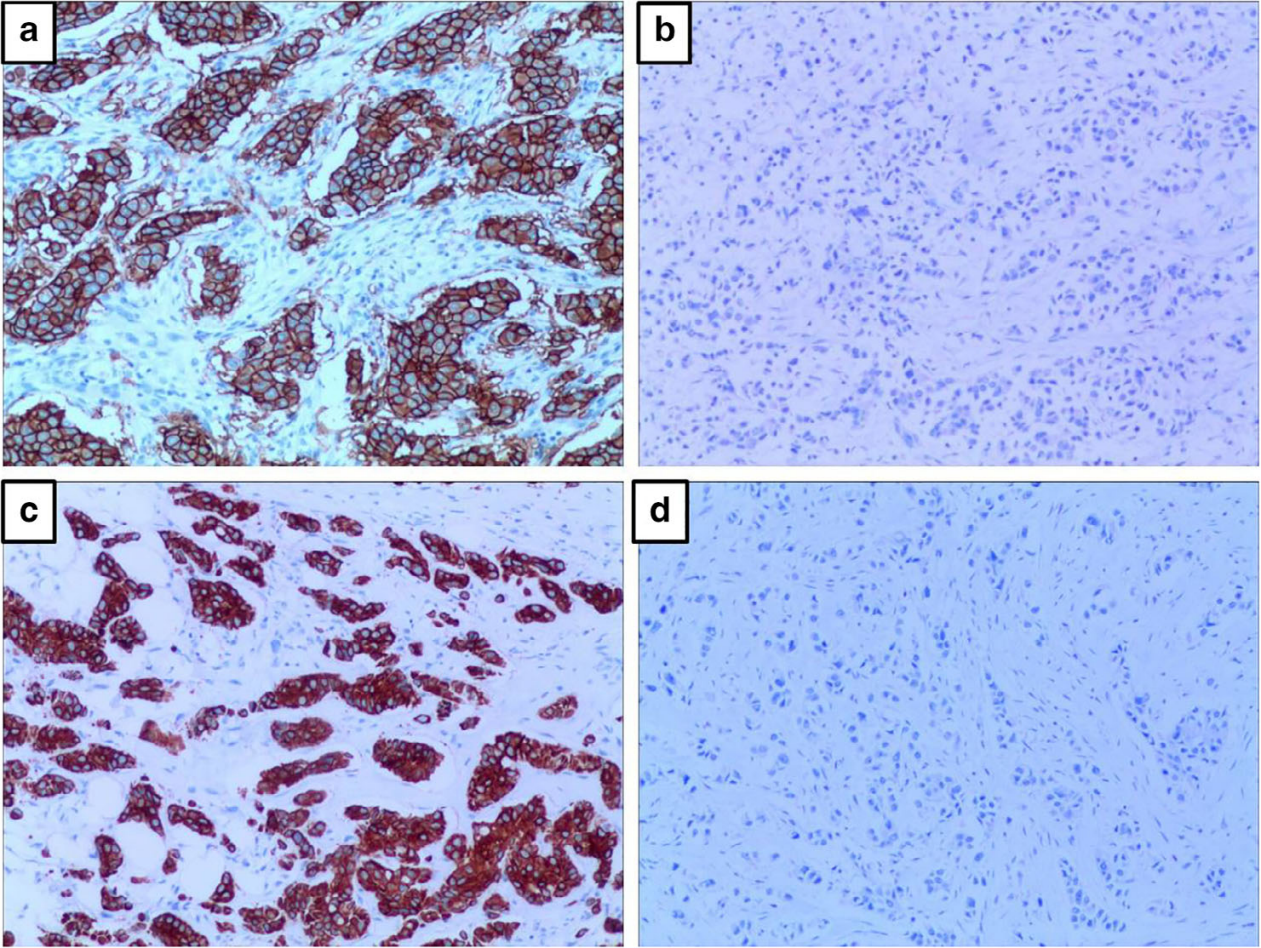
Ezrin expression in breast cancer examined by immunohistochemistry assay. (**a**) Ezrin expression was localized in cancer cell membrane ×100; (**b**) Ezrin expression was negative in the cell membrane ×100; (**c**) Ezrin expression was positive in the cytoplasm of breast cancer cells ×100; (**d**) Ezrin expression was negative in the cytoplasm of breast cancer cells ×100.

The ezrin (coded by *EZR* gene) expression was positive with uniform brown‐yellow granules in the cell membrane, cavity surface and cytoplasm of the cancer cell in breast cancer patients (Fig 10 **)**. Of the 120 cancer samples, ezrin expression was positive in 98 cases and negative in 22 cases. No correlation was found between ezrin expression site and patients’ clinicopathological features.

## Discussion

Ezrin protein (coded by EZR) is a member of the ERM (ezrin‐radixin‐moesin) protein family.^13^ Its function is to connect cell surface receptors, especially adhesion molecules and actin cytoskeleton.^14^, ^15^ Ezrin is mainly expressed at the top of cell surface and participates in maintaining the polarity of epithelial cells.^16^, ^17^, ^18^ Recent studies have found that ezrin participates in cells and bases interaction by regulating adhesion molecules and signal transduction pathways which may play an important role in the invasion and metastasis of cancer cells.^19^, ^20^

The role of ezrin is to change the infiltration potential of cancer cells, which can be divided into two aspects: (i) Forming complex with calyx glycoprotein to play an anti‐adhesion role thereby reducing the adhesion between cancer cells, and (ii) activating calyx glycoprotein to participate in the remodeling of cytoskeleton, forming pseudopodia and enhancing the migrational ability of cells. Studies have shown that ezrin expression in tumors was significantly increased compared to corresponding normal tissues.^21^, ^22^ At the same time, the location of ezrin in tumor cells also changed when compared to normal cells. In normal cells, ezrin was mainly expressed in actin‐rich microvilli, pseudopodia and other surface structures. However, in tumor cells, ezrin expression was positive in cytoplasm and cell membrane.^23^ A study in pleomorphic adenoma of the salivary gland showed that ezrin not only enhanced cell proliferation, but also promoted malignant transformation of pleomorphic adenoma.^24^ The expression level of ezrin in hepatocellular carcinoma cell lines was consistent with its invasive ability, and transfection of antisense oligonucleotides could significantly inhibit the invasive ability of cells.^25^ In addition, ezrin was also associated with metastasis of primary osteosarcoma according to the previous study.^26^ Approximately 55.7% of nonmetastatic osteosarcoma cases express ezrin, and the prognosis of ezrin‐positive expression osteosarcoma cases was poor compared to negative cases.^26^ Detection of ezrin expression in colorectal cancer showed that the expression of ezrin in colorectal cancer was significantly higher than that in normal colorectal tissues, and the expression of ezrin was correlated with the degree of differentiation, lymph node metastasis and Dukes stage.^27^

In our present work, we have investigated the *EZR* gene expression in breast cancer, biological function, pathway and correlation with the prognosis through bioinformatics analysis and immunohistochemistry assay. We found that the *EZR* gene was upregulated in cancer tissue compared to normal breast tissue in breast cancer patients. This may indicate that EZR could play an important role in the development of breast cancer. Furthermore, we investigated the ezrin protein coded by *EZR* gene in 120 samples of breast cancer tissue by immunohistochemistry assay and found that the ezrin positive expression in 98 cases was present in 81.7% of all the cases. The *EZR* and relevant genes were mainly enriched in signaling receptor binding, cell adhesion molecule binding, leukocyte transendothelial migration, proteoglycans in cancer, regulation of actin cytoskeleton pathway. Survival analysis of the present work showed that high expression of EZR mRNA was correlated with poor overall survival of the breast cancer patients. However, the disease‐free survival was not associated with the *EZR* expression level.

In conclusion, EZR mRNA was upregulated in breast cancer which was in accordance with its coding protein (ezrin) expression pattern detected by immunohistochemistry assay. The upregulated EZR mRNA and protein can be used as potential biomarker for overall survival in the future.

## Disclosure

The authors confirm that there is no conflict of interest.
